# Management of Biliary Cysts in Adults: A Single-Center 10-Year Experience

**DOI:** 10.7759/cureus.37964

**Published:** 2023-04-21

**Authors:** Deepak Ranvir, Asma Khalife, Rajeshkumar C Mahey, Bhushan Telang, Gagan Soni, Abhi H Kothari, Rajeev Joshi

**Affiliations:** 1 Department of General Surgery, H.B.T. Medical College & Dr. R.N. Cooper Municipal General Hospital, Mumbai, IND; 2 Department of General Surgery, Topiwala National Medical College & B.Y.L. Nair Charitable Hospital, Mumbai, IND; 3 Department of Surgical Oncology, Government Medical College & Hospital, Aurangabad, IND; 4 Department of Surgery, All India Institute of Medical Sciences, New Delhi, IND; 5 Department of General Surgery, K.J. Somaiya Medical College & Research Centre, Mumbai, IND

**Keywords:** biliary tree, malignant, jaundice, anomalous pancreaticobiliary duct union, biliary tree diseases, bilioenteric anastomosis, biliary cyst, choledochal cyst

## Abstract

Background: A choledochal cyst is a cystic dilatation of the biliary tree, also termed a biliary cyst, including an intrahepatic cyst as well. Magnetic resonance cholangiopancreatography (MRCP) is the gold standard investigation of choice for this pathology. Todani classification is most commonly used to classify choledochal cysts.

Materials and methods: A total of 30 adult patients with choledochal cysts presenting at our center from December 1, 2009, to October 31, 2019, were studied retrospectively.

Results: The mean age was 35.13 years ranging from 18 to 62 years with a male-to-female ratio of 1:3.29. Of the patients, 86.6% presented with abdominal pain. Total serum bilirubin was raised in six patients with a mean of 1.84 mg/dL. MRCP was done in all patients, which had almost 100% sensitivity. Two cases had anomalous pancreaticobiliary duct union. In our study, we found only type I and type IVA cysts according to the Todani classification (type IA = 56.3%, IB = 11%, 1C = 16%, and IVA = 17%). The mean size of the cyst was 2.37 cm. Complete cyst excision with Roux-en-Y hepaticojejunostomy was performed in all patients. Four patients had surgical site infections and two had bile leaks. One patient developed hepatic artery thrombosis. All complications were eventually managed conservatively. Mortality was nil in our study with the mean postoperative stay being 7.97 days.

Conclusion: Adult presentation of biliary cysts is not an uncommon entity in the Indian population and should be considered as a differential diagnosis of biliary pathology in adult patients. Complete excision of cysts with bilioenteric anastomosis is the current treatment of choice.

## Introduction

Choledochal cysts are rare congenital dilatations of the biliary tree. Choledochal cysts were first described in 1723 by Vater and Ezler with an incidence of 1:100,000 live births in Western countries, reaching 1:1000 in Asia, mostly reported in Japan. It is a disease of children with nearly 80% diagnosed in the first decade of life, and the rest present or are incidentally detected in the adult age group. Anomalous pancreaticobiliary duct union (APBDU) proposed by Babbitt in 1969 is the most widely accepted hypothesis for this pathology, which can be demonstrated by magnetic resonance cholangiopancreatography (MRCP), which is also the investigation of choice [1-4]. The historical classical triad of choledochal cysts is intermittent abdominal pain, jaundice, and palpable right upper quadrant mass, not commonly observed nowadays due to the wide availability of imaging modalities [5]. Todani modification (1977) of the Alonso-Lej classification of choledochal cysts is the most widely used classification, which divides the cysts into eight alphanumerical subtypes [3].

## Materials and methods

Our study was designed as a retrospective analytical study of 30 adult patients with choledochal cysts operated on in the Department of General Surgery at Topiwala National Medical College & B.Y.L. Nair Charitable Hospital from 1st December 2009 to 31st October 2019. The ethics committee's approval was taken. All patients above the age of 18 years with a diagnosis of a choledochal cyst were included in this study. Pregnant females were excluded from this study. Patient demographics, clinical presentation, blood and imaging investigations, previous treatment history, operative findings, postoperative outcomes, and follow-up data were obtained from medical records and analyzed. All imaging studies were retrospectively reviewed again from our imaging database especially to confirm the presence or absence of APBDU to alleviate reporting bias. In all cases, the diagnosis was confirmed by histopathological examination of the surgical specimen. Cysts were classified according to the Todani classification based on MRCP. Postoperative complications were defined as "early" if occurred within 30 days and "late" if occurred after 30 days and classified using the Clavien-Dindo classification [6].

## Results

A total of 30 patients were included in this retrospective study. The mean age was 35.13 years ranging from 18 to 62 years. Female predominance was observed with a male-to-female ratio of 1:3.29.

Out of 30 patients, two were detected incidentally in imaging done for other pathologies, 26 (86.6%) patients presented with abdominal pain, and among these, six (20%) patients had jaundice, and one patient presented with cholangitis for which initial conservative management with endoscopic retrograde cholangiography (ERCP) with stenting followed by operative management was done. Two patients had only dyspepsia and bloating as a presentation. In our series, none of the patients presented with a classical triad of abdominal pain, jaundice, and palpable right upper quadrant mass.

Total serum bilirubin was raised in six patients with a mean of 1.84 mg/dL (range = 0.3-12.5 mg/dL). Transabdominal ultrasonogram (USG), which was used as the initial investigation, showed the dilated biliary system in 21 (70%) patients. MRCP was done in all patients to characterize the cysts and other pathologies associated. APBDU was found in only two (6.6%) cases.

In our study, we encountered only type I (83.3%) and type IVA cysts, according to the Todani classification. Type IA cysts were commonly found in 56.3% of patients, IB in 11%, IC in 16%, and type IVA in 17% of patients. The mean size of the cyst was 2.37 cm. Cystolithiasis was found in 10 patients, among which four had biliary obstruction, which was relieved by ERCP-guided ductal clearance, and hepatolithiasis in two, which were cleared during surgery.

All the patients underwent complete excision of choledochal cyst with Roux-en-Y hepaticojejunostomy, and one patient was operated on by minimally invasive technique. Histopathological examination was done for all surgical specimens to confirm the diagnosis and to look for malignancy. None of the specimens showed dysplasia/malignancy.

Postoperative complications based on the Clavien-Dindo classification were seen in eight patients, as shown in Table 1. Postoperatively, four patients had superficial surgical site infections managed conservatively and two patients had bile leaks from bilioenteric anastomosis. Both of them were managed conservatively. One patient developed right hepatic artery thrombosis leading to ischemic hepatic injury, which was also managed conservatively with anticoagulants. No death was observed. The mean postoperative stay was found to be 7.97 days ranging from six to 13 days.

**Table 1:** Postoperative complications

Clavien-Dindo grade	Number of patients
I	5
II	2
III	1

## Discussion

Choledochal cysts are also termed biliary cysts, including intrahepatic cysts also. Biliary cysts are defined as cystic dilations involving the biliary tree at single or multiple segments of both the extrahepatic as well as intrahepatic bile ducts. Its incidence is higher in Japan and Korea (1:1000 live births) compared with Western countries (1:100,000-1:150,000 live births). Incidence in the Indian subcontinent is not reliably known due to the paucity of data. Though the disease is classically regarded as a condition of children, currently it was more often observed in adult females signifying the shift in presentation from the first decade of life to adulthood and improvement in diagnostic high-resolution cross-sectional imaging modalities. Worldwide, studies show female predominance consistent with other biliary system disorders with a female-to-male ratio varying from 3:1 to 4:1, which was consistent with our study [2-4,7,8].

Biliary cysts present with a wide range of symptoms ranging from asymptomatic to spontaneous perforation, cholangitis, and pancreatitis. According to the studies, adult patients had more severe symptoms due to the prolonged presence of the pathology. The commonest presentation in adults is abdominal pain, which was also contemplated in our study. The classical triad typically described is found less commonly (0-17%) nowadays due to improvements in diagnostic modalities [1,4,5]. In our study, none of the patients had the classical triad and two patients were detected incidentally.

USG is often used as an initial diagnostic tool and has a sensitivity of 71-97% in detecting biliary cysts, but it has many setbacks due to observer variation, obscuration by bowel gases, and difficulty in differentiating gall bladder from cysts. In our study, 70% of patients were diagnosed with the dilated common bile duct on primary USG. MRCP is the gold standard investigation for diagnosing and classifying biliary cysts with a sensitivity reaching 100% [2,4,5]. All of our patients underwent MRCP for detailing cyst anatomy and planning for surgery.

The exact etiology of biliary cysts is unknown, and various hypotheses had been proposed. Till now, the commonly accepted hypothesis of APBDU by Babbitt postulated long common biliopancreatic channel (>15 mm) leads to the mixing of biliary and pancreatic secretion, which in turn activates the pancreatic enzymes leading to the weakening and dilatation of the bile duct. The presence of APBDU increases the risk of malignancy. This hypothesis is supported by many series and experimental animal studies but fails to explain the predominant intrahepatic cysts formation and some studies report low incidence (14-44%). Other reported hypotheses include oligoganglionosis of the biliary tree, partial biliary obstruction, ductal plate malformation, and sphincter of Oddi dysfunction [3,9,10]. In our study, we found APBDU in 6.6% of the patients only.

According to the literature, type I biliary cysts are commonly encountered with an incidence reaching up to 90%, followed by type IV with an incidence of 15-35%. Our study also contemplates the same, but in our study, type IA was the commonest type, which is discordant with the literature. Treatment of the biliary cysts varies according to the type of the cyst. Historically, cyst-enterostomy was considered as surgery of choice for biliary cysts, but later studies showed a high recurrence of symptoms and risk of malignant transformation of the remaining cyst wall. At present, complete excision of the cyst with bilioenteric anastomosis is the surgery of choice. The first documented resection of a biliary cyst was by MacWorter in 1924. Roux-en-Y hepaticojejunostomy/choledochojejunostomy is the preferred approach either open or minimally invasive [1,9,11]. The same was done in our study with one patient treated by a minimally invasive technique.

Morbidity following surgical treatment was reported between 13% and 40%. The majority of complications were wound-related, with anastomotic leaks followed by pancreatic fistula in the early phase. In the late phase, anastomotic strictures, cholangitis, adhesive intestinal obstruction, and malignancy were reported [10,12]. The risk of malignancy in biliary cysts exists even after the complete surgical excision, as reported in Korean and Japanese studies, but it was virtually nil in Indian studies so far, which can be attributed to the low incidence of APDBU, complete excision of cyst rather than drainage procedures, and shorter long-term follow-up [10]. In our study, the early complication rate was 23.3% and no late complications were recorded with a median follow-up of 25 months.

Limitations of the study

This study had a small sample size of 30 patients and a shorter postoperative follow-up duration. A study with a larger cohort and a longer median postoperative follow-up duration is warranted to validate these findings.

## Conclusions

Adult presentation of biliary cysts is not an uncommon entity in the Indian population, and it should be considered a differential diagnosis of biliary pathology in adult patients. Complete excision of cysts with bilioenteric anastomosis is the current treatment of choice, which can be safely done with minimally invasive techniques. A low frequency of postoperative complications and malignancy was seen in a one-year follow-up in our study. However, further validation of these results with long-term prospective trials with a larger cohort is imperative.
